# Cold atmospheric plasma, a novel promising anti-cancer treatment modality

**DOI:** 10.18632/oncotarget.13304

**Published:** 2016-11-11

**Authors:** Dayun Yan, Jonathan H. Sherman, Michael Keidar

**Affiliations:** ^1^ Department of Mechanical and Aerospace Engineering, The George Washington University, NW, Washington, DC, USA; ^2^ Neurological Surgery, The George Washington University, NW, Washington, DC, USA

**Keywords:** cold plasma, cancer treatment, reactive species, selectivity

## Abstract

Over the past decade, cold atmospheric plasma (CAP), a near room temperature ionized gas has shown its promising application in cancer therapy. Two CAP devices, namely dielectric barrier discharge and plasma jet, show significantly anti-cancer capacity over dozens of cancer cell lines in vitro and several subcutaneous xenograft tumors in vivo. In contrast to conventional anti-cancer approaches and drugs, CAP is a selective anti-cancer treatment modality. Thus far establishing the chemical and molecular mechanism of the anti-cancer capacity of CAP is far from complete. In this review, we provide a comprehensive introduction of the basics of CAP, state of the art research in this field, the primary challenges, and future directions to cancer biologists.

## PLASMA AND COLD PLASMA

**Figure 1:**
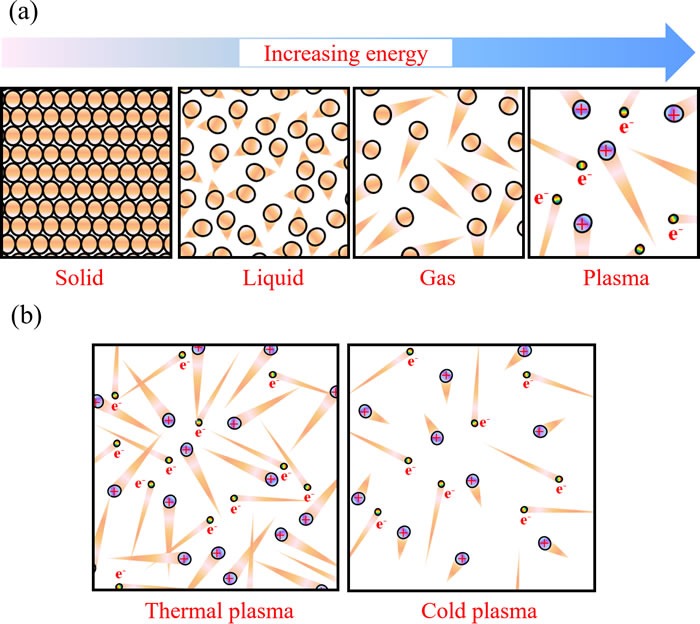
The physical description of plasmas **a**. Schematic illustration of the four fundamental states of matter. The triangular tails represent the thermal motion strength of particles. **b**. Schematic illustration of the thermal plasma and the cold plasma. Brown balls, violet balls, and iridescent balls represent the neutral atoms, the positively charged ions, and electrons, respectively.

There are four fundamental states of matter: solid, liquid, gas, and plasma (Figure 1a). As the energy exerting on atoms increases, the thermal motion of atoms in the solid aggravates and finally overcomes the restrictive interaction between atoms in solid such as ionic bond and forms liquid. Similarly, when the atoms in liquid obtain adequately large energy to overcome the restrictive Van der Waals force from surrounding atoms, these liquid atoms will transfer into gas atoms. Obviously, the translational energy of atoms in gas is much larger than that in liquid and in solid. When the energy is large enough for the electron to overcome the electrostatic potential barrier, the electron will be stripped away creating a free electron and a positive charged ion. This process is called ionization. The plasma is on avarage a neutral ionized gas composed of positive charged ions, electrons, and neutral particles.

In general, temperature increases when matters transform from solid to liquid to gas and to plasma. The temperature of plasma is determined by thermal motions of electrons and heavy particles such as atoms and ions. In the case of a common thermal plasma, when the density of particles is high, due to intensive collisions between eletrons and heavy particles, all particles approach thermal equilibrium [1]. The temperature in such plasma is high, over several thousand degrees [1]. These plasmas are typically used under the atmospheric pressure conditions. On the other hand, if atmospheric pressure plasma discharge is fast, there is another class of plasmas in which electrons and heavy particles are in thermal non-equilibrium. In this case, temperature of the heave particles is much lower than that of the electrons (Figure 1b). We shall call these plasmas, cold atmospheric plasmas (CAP). The heavy particle temperature of CAP is between 25°C and 45°C [2]. Such plasmas can be used in biomedicine [3]. Many reactive species including oxygen-based radicals, nitrogen-based radicals, and other components are generated in CAP [4–6]. This complicated chemistry leads to a myriad of interaction between CAP and biological systems including cells and tissues [7–9].

## CAP DEVICES

**Figure 2:**
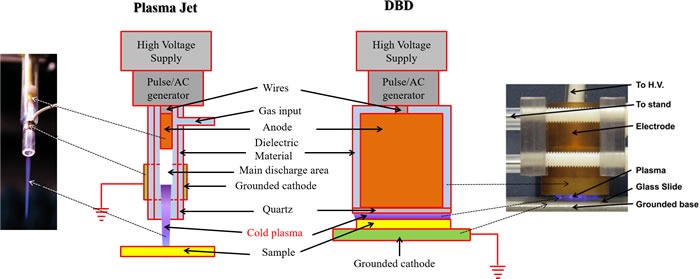
The plasma jet device and dielectric barrier discharge (DBD) device are two main CAP devices used in plasma medicine The same components in the plasma jet and DBD are drawn with the same colors. The left inset is reproduced with permission from Alan Siu, *et al*., PLoS ONE, 10(6), e0126313 (2015). Copyright 2015 Public Library of Science. The right inset is reproduced with permission from Sameer Kalghatgi, *et al*., PLoS ONE, 6(1), e16270 (2011). Copyright 2011 Public Library of Science.

Two main approaches have been widely used to generate CAP, namely direct and indirect discharges. In an indirect discharge, the active plasma species are transported by a gas flow from the main discharge arc. In a direct discharge, living tissue or cells is one of the electrodes and is an active part of the discharge. Based these principles, two CAP devices, the plasma jet [6, 10, 11] and the dielectric barrier discharge (DBD) [12, 13], have been developed and widely used in plasma medicine [14–22]. The plasma jet is also called the plasma pencil [23, 24], the plasma needle [2, 25, 26], or the plasma gun [27] in some references. As shown in Figure 2, the plasma jet device and the DBD device share similar physical principles, components, and materials. In these two devices, a violet plasma is generated between an annode and a cathode. Either anode or cathode is covered by a layer of dielectric materials such as quartz [19, 28]. In many plasma jet devices, the quartz hollow tube is used as the dielectric layer on the cathode [2, 29]. The metal cathode such as copper surrounds the quartz tube. In addition, the plasma jet device needs a carrying gas such as helium [18, 26, 30] or argon [25, 31, 32] to sustain the formation of CAP while the DBD device can generate the plasma directly in the air [20–22, 33]. In some applications, oxygen [4, 34] and nitrogen [35, 36] have been added in the carrying gas to achieve the specific chemical composition. Due to the continuous flow of the carrying gas, a CAP jet forms. On the other hand, the DBD device tends to generate a short but a wide plasma. Moreover, the functions of samples in the two cold plasma devices are also different. In the plasma jet device, the sample is just treated by the plasma jet [4, 18, 37]. On the other hand, in DBD device, the sample is a part of discharge [20, 22, 38]. The CAP in DBD will not be generated if the sample is not adequately close to the second electrode. Based on these properties and features, the plasma jet device may be more suitable for gentlly treating a small area on a sample. In contrast, the DBD may be more suitable for a more intense treatment on a large area of sample. A recent review written by X. Lu, *et al*., comprehensively introduced the physical foundation of the reactive species generation in different CAP devices [39].

## RESEARCH STATUS

**Figure 3:**
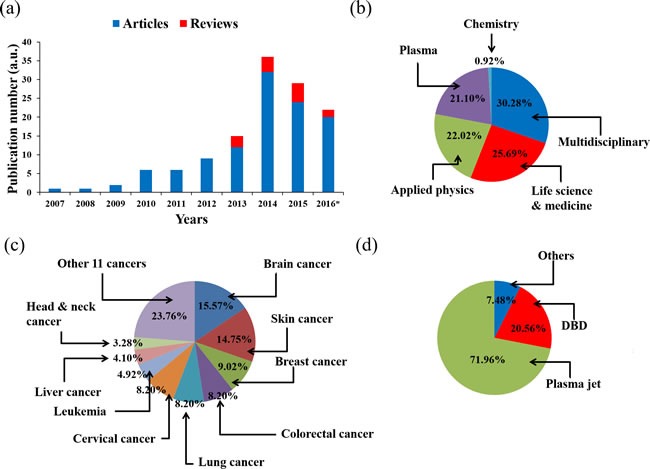
The research status of the application of CAP on cancer treatment by 2016 **a**. Publication number. *: by the end of September. **b**. The journal types of articles. **c**. Cancers in articles. **d**. Plasma devices in articles.

There exist a number of published reviews that discuss the application of CAP on cancer treatment [40–44] [45]. In this review, we focused on introducing the basic concept of CAP and the biological basis of the anti-cancer mechanism of CAP. Since the first report about the killing effect of DBD on melanoma in 2007, the field of CAP application in cancer treatment experienced a fast growth (Figure 3a). Through a comprehensive survey of all publications by September 2016, it is found that about 75% papers in this field were published in the multidisciplinary journals such as PLoS ONE, the applied physics-related journals such as Applied Physics Letters, as well as the plasma-related journals such as Plasma Processes and Polymers (Figure 3b). On the other hand, only about 25% papers were published in the life science & medicine-related journals. As a result, the research in this field is mainly focused on describing the anti-cancer effect of CAP treatment on different cancer cell lines [41, 44] and tumors in animal models [20, 30, 46]. To date, the CAP treatment has demonstrated its significant anti-cancer capacity over approximately 20 cancers types *in vitro*. Among these cancer cell lines, brain cancer [27, 47, 48], skin cancer [2, 19, 49], breast cancer [50–52], colorectal cancer [15, 53, 54], lung cancer [18, 46, 55], cervical cancer [56–58], leukemia [23, 59, 60], hepatoma [25, 37, 58], as well as head & neck cancer [61–63] have been intensively investigated (Figure 3c). Moreover, about 70% of the entire publications employed the plasma jet devices as the anti-cancer tools (Figure 3d).

In addition to resisting the growth of cancer cells, CAP is also able to restore the sensitivity of chemo-resistant cancer cells to specific drugs. One example is that CAP restored temozolomide (TMZ)-resistant glioblastoma cells to TMZ therapy [64]. Another example is that CAP made tumor necrosis factor-related apoptosis-inducing ligand (TRAIL)-resistant colorectal cancer cells sensitive to the TRAIL treatment [65].

Moreover, CAP can obtain a stronger anti-cancer capacity through the synergistic application with nanoparticles technologies. M.G. Kong, *et al*. gave a detailed illustration in a review to describe the potential synergistic application of CAP and nanoparticles in medicine [66]. The enhanced anti-melanoma effect was first achieved using CAP to treat melanoma cancer cells which had been pretreated with the anti-FAK antibody-conjugated gold nanoparticles [49]. Clearly, such surface-modified nanoparticles weaken the normal function of FAK, which may intensify the CAP-triggering detachment of melanoma cells from the substrate. It was further demonstrated that the pretreatment of gold nanoparticles without the specific antibody modification also enhanced the anti-glioblastoma effect of the plasma jet [67]. A recent study demonstrated that the combined treatment of polyethylene glycol (PEG)-coated gold nanoparticles and CAP increased cancer cells death in solid tumors and decreased epithelial-mesenchymal transition (EMT) [68]. In addition to using nanoparticles, drug encapsulated core-shell nanoparticles synthesized *via* co-axial electrospraying has also shown its synergistic anti-cancer potential with CAP on breast cancer cells [69]. These studies indicate that nanoparticles may weaken or damage the normal function of specific proteins or pathways, which resist the intracellular change due to the CAP-originated reactive species.

**Figure 4:**
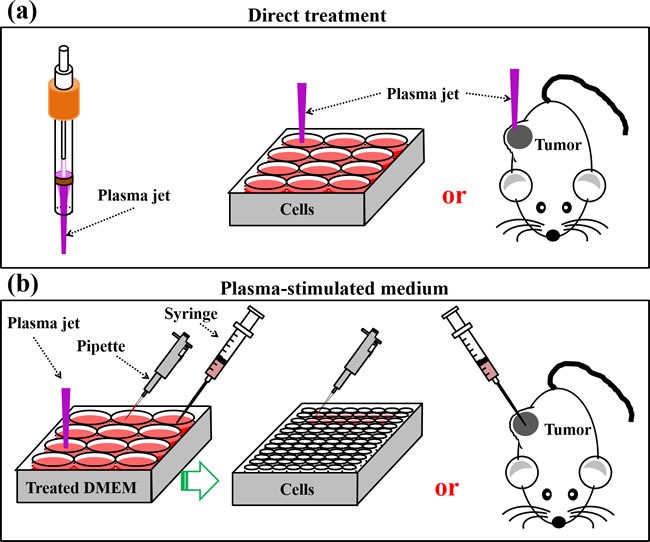
Two basic strategies to use CAP **a**. Direct CAP treatment on cancer cells *in vitro* or on subcutaneous xenografted tumors *in vivo*. **b**. Indirect CAP treatment on solutions mainly medium. These PSM will be used to inhibit the growth of cancer cells seeded in multi-wells plate or the tumor tissues in mice.

Two basic strategies of using CAP have been developed. One is to employ the plasma jet [25] or DBD [19] to directly treat the cells seeded in a petri dish or a multi-wells plate or the subcutaneous tumors in mice (Figure 4a). Another approach is to use the cold plasma-stimulated solutions (PSA) mainly the cold plasma-stimulated medium (PSM) to inhibit the growth of cancer cells during the standard cell culture process [47, 70] or to inhibit the growth of tumor tissues by injecting PSM into the tumor tissues of mice [71] (Figure 4b). PSM is also named cold plasma-activated medium (PAM). Most studies utilized the first method. Over past 3 years, the second approach is gradually becoming a hot topic [66, 72–80]. In this review, we just discussed the former one.

## INTERACTION BETWEEN CAP AND BIOLOGICAL SYSTEM

**Figure 5:**
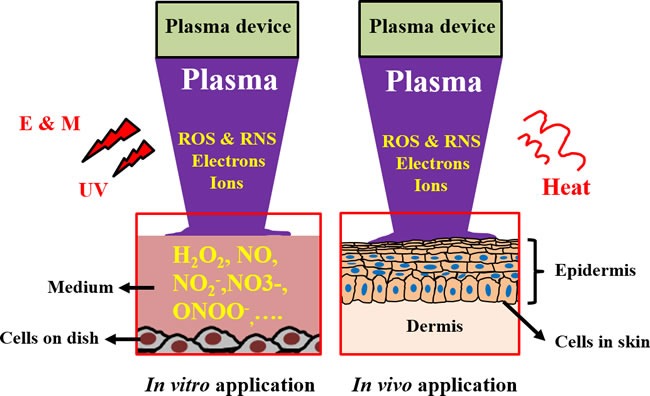
Schematic illustration for the interaction between CAP and cells ***in vitro*** and ***in vivo***. Abbreviations: E & M: electromagnetic field, UV: ultraviolet. The dissolved reactive oxygen/nitrogen species in the culture medium have been regarded as the main factor causing the death of cancer cells *in vitro*. However, the inhibited growth of subcutaneous tumor tissues through the treatment of CAP above the skin is still a puzzled question in plasma medicine.

The interaction between CAP and cells in tissue or cells grown in a petri dish builds the foundation of the anti-cancer effect of CAP. Such interaction is a combination of physical and chemical factors as shown schematically in Figure 5. Ultraviolet, heat, and electromagnetic field are physical factors in CAP. Chemical factors include dozens of reactive species that are generated in the gas phase of CAP. Among them, oxygen-based species such as hydroxyl (OH.) [5, 18, 50], singlet oxygen (^1^O_2_) [5, 35, 50], superoxide (O_2_.^-^) [5], hydrogen peroxide (H_2_O_2_) [5], ozone (O_3_) [5, 60], as well as nitrogen-based species such as nitric oxide (NO) [5, 18, 36], nitrogen dioxide (NO_2_) [60], nitrogen trioxide (NO_3_) [60], nitrous oxide (N_2_O) [60], and dinitrogen tetroxide (N_2_O_4_) [60] have been observed in CAP. In addition, positive charged ions such as N_2_^+^ [18, 37, 50] and electrons [5] are also generated by CAP.

To date, most *in vitro* studies have focused on the anti-cancer effect of CAP on cancer cells cultured in a petri dish or in a multi-well plate. When the CAP treatment performs, a layer of cell culture medium always covers the cancer cells. Thus, the above-mentioned physical and chemical factors will touch the medium first (Figure 5). The effect of ultraviolet, heat, as well as electromagnetic field on the medium is negligible. The slight increase in the temperature of medium after CAP treatment from room temperature to about 35°C is the only confirmed evidence of the physical effect of CAP on the medium [33, 81]. The final temperature of the medium is determined on its initial temperature, composition, the CAP treatment dose and the power of discharge [33, 81]. Because the standard temperature in the incubator is 37°C, the slightly warmed CAP-treated medium clearly will not inhibit the growth of cancer cell. In addition, ultraviolet and electrons in DBD have been proved to be negligible factors contributing to the intracellular DNA damage triggered by CAP [5]. Thus, the observed cellular response to CAP treatment *in vitro* may be mainly due to various CAP-originated reactive species [9]. This may be the substantial difference between CAP and some conventional chemotherapy methods. The later may also generate intracellular ROS stress by different intracellular pathways. However, CAP itself is a tunable source of reactive species. The intracellular ROS stress is due to the specific combination of the CAP-originated species.

Despite the understanding on the solvation of plasma component into the aqueous solution is far from clear, two trends are observed. First, most components of the medium, in particular, amino acids dissolved in medium [82] and the amino acids in proteins [70] are modified after the CAP treatment. Among 20 amino acids, cysteine shows the strongest reactivity towards the plasma-originated reactive species dissolved in PSM though the mechanism is still remain disputable [70]. Second, several oxygen reactive species (ROS) such as H_2_O_2_ [33, 56, 83], and nitrogen reactive species (RNS) such as NO_2_^-^ [70, 84, 85] and ONOO^-^ [86] have been widely detected in PSM or other plasma-treated aqueous solutions. In addition, many authors conclude that NO has been formed in PSM [17, 56, 84]. However, only the concentration of NO_2_^-^ rather than directly NO [17, 52, 81] was measured. The NO_2_^-^ detected in medium may not be simply due to the oxidation of NO in medium, though it is the sole explanation currently to explain the slow increase of NO_2_^-^ in PSM during the storage [70, 85] . It is plausible to assume that NO_2_^-^ is formed as result of the unknown interaction between medium and CAP. Thus, though the existence of NO in gas phases of CAP has been widely recognized [5, 18, 36], the generation of NO in PSM has not been directly proven.

## THE ANTI-CANCER EFFECT OF CAP IN VIVO

**Figure 6:**
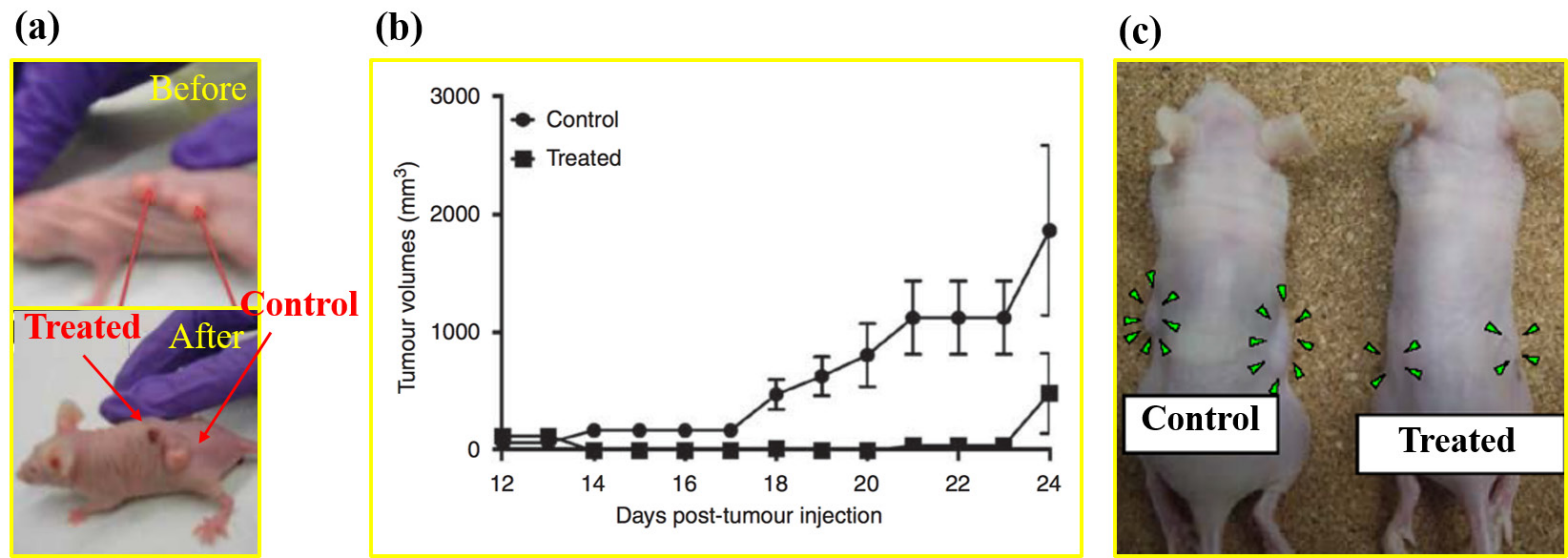
The anti-cancer effect of CAP in mice model (**a**) Image of mouse with two tumors before and after the plasma jet treatment for 24 hr. The subcutaneous tumors are grown from the seeded bladder cancer cells (SCaBER) [46]. Reproduced with permission from M. Keidar, *et al*., British Journal of Cancer, 105, 1295 (2011). Copyright 2011 Cancer Research UK. (**b**) Cold plasma treatment effect on the growth of established tumor in a murine melanoma model [46]. Reproduced with permission from M. Keidar, *et al*., British Journal of Cancer, 105, 1295 (2011). Copyright 2011 Cancer Research UK. (**c**) Images of nude mice bearing subcutaneous NOS2TR tumors before and after the injection of the cold plasma-stimulated medium. Green arrowheads indicate tumor site [71]. Reproduced with permission from F Utsumi, *et al*., PLoS ONE, 8, e81576 (2013). Copyright 2013 Public Library of Science..

To date, several investigations have used CAP to treat subcutaneous xenograft tumors [20, 27, 30, 62, 87, 88] and melanoma in mice [89, 90]. All those studies achieved a similar result that the growth of tumor *in vivo* was significantly halted by the CAP treatment on the skin of mice. For example, our study on a bladder tumor mouse xenograft through subcutaneous injection displays this effect [46] (Figure 6a). After 24 hours following a 2 minutes of CAP treatment, the treated tumor significantly decreased in size and could not been observed on the skin of mice [46]. We have performed similar experiments on a murine melanoma model and found that tumor growth was completely inhibited over 3 weeks after the CAP treatment [46] (Figure 6b). Corresponding mice survival rates were also strongly increased compared with the control group without the CAP treatment [46]. A very recent study on the anti-melanoma effect of DBD further confirmed that the CAP-treated melanoma tissue completely disappeared at the 22rd day after the treatment [90].

The anti-cancer mechanism of CAP treatment *in vivo* is still an open question. Very recently, N. Gaur, *et al*., proved that H_2_O_2_, NO_2_^-^, and OH. could be generated in the phosphate buffered saline (PBS) covered by a 1 mm thick gelatin film which was directly touched by the plasma jet [91]. This result indicates that the diffusion of reactive species across the skin analogue is possible. However, other explanations have also been proposed. One promising candidate is that the CAP treatment activates the immune response *in vivo* to attack the tumor [92–94]. The macrophages can be activated *in vitro* by a uniform nanosecond pulsed DBD (nspDBD) and improves the healing effect at an artificial wound [94]. The uniform nspDBD also enhanced the anti-tumor effects through both the induction of immunogenic cell death in tumor cells and augmentation of macrophage's function [93]. Vandana Miller, *et al*. provided a comprehensive introduction about the promising cancer immunotherapy based on the CAP treatment in a recent review [92]. By optimizing the parameters of CAP to induce immunogenic cell death in tumors locally, it is possible to trigger specific, protective immune responses systematically [92]. In addition to these direct CAP treatments, the anti-cancer capacity of PSM *in vivo* has also been confirmed by directly injecting PSM into the subcutaneous xenograft tumors in mice (Figure 6c) [71].

Recent *in vivo* studies in a melanoma mice model demonstrated that reactive species such as ROS and RNS are the main factors contributing to the elimination of tumor by CAP [90]. However, it should be noted that the mechanism of action for CAP *in vivo* might be different from one *in vitro*. It was observed that just using the gel/H_2_O_2_ mixture could not generate a tumor killing efficacy as significant as that of DBD on the melanoma in a mouse model [90]. In fact, the application of the gel/H_2_O_2_ mixture just slowed the tumor growth, but did not eliminate the tumor [90]. Reactive species other than H_2_O_2_ may also be involved in the interaction between CAP and the cancerous tissue.

Several comments should be made regarding the physical factors such as radiation, thermal effect, as well as the electromagnetic field. Despite these physical factors being regarded as minor players for the anti-cancer effect of CAP in *in vitro* conditions, some effects might be expected *in vivo* when CAP directly treats skin or epithelial tissues. One recent study using a mouse xenograft model demonstrated that the increase of temperature on the CAP-treated tumor tissue *in vivo* is negligible [90] confirming previous reports [46]. Though the contribution of electric field generated by CAP to the anti-cancer capacity of CAP was found to be small [90], further study of this effect and its importance is warranted [95]. Overall, the effect of physical and chemical factors on the *in vivo* application is still largely unknown.

## THE ANTI-CANCER MECHANISM OF CAP IN VITRO

### The chemical essence of the toxicity of CAP

In most *in vitro* studies, cancer cells were immersed in a layer of cell culture medium during the CAP treatment [62, 96, 97]. As we mentioned above, this thin medium layer facilitates the transition of reactive species in gas phase into the reactive species dissolved in liquid phase [98]. Thus, the change in the CAP treated medium or PBS is the foundation to understand the chemical essence of CAP's toxicity *in vitro*. Due to the existence of buffering chemicals in medium or in PBS, short time CAP treatment just causes a negligible change in pH of medium or PBS [33, 81]. Among diverse plasma-originated species, H_2_O_2_ has been proved to the main anti-cancer reactive species causing the death of cancer cells *in vitro* [37, 56, 72, 73, 75–77, 81, 83, 99, 100]. Actually, the medium with adequately high concentration of H_2_O_2_ is toxic to various cells including cancer cells [101, 102]. But, the CAP treatment is not simply equal to the H_2_O_2_ treatment even just in *in vitro* studies. Synergistically using H_2_O_2_/NO_2_^-^ or H_2_O_2_/NO_2_^-^/NO_3_^-^ in cell culture medium or PBS can generate an anti-cancer effect more close to that generated by the CAP treatment than just using the H_2_O_2_ containing medium or PBS [79, 103]. Nonetheless, RNS is a minor anti-cancer factor for *in vitro* studies [79, 103]. We have found that even the concentration of nitrite in medium increases to be 100 times stronger than that generated by the CAP treatment, the growth of glioblastoma cells, breast cancer cells, as well as pancreatic cancer cells will not be noticeably affected (unpublished results).

### The change of cellular membrane and shape

**Figure 7:**
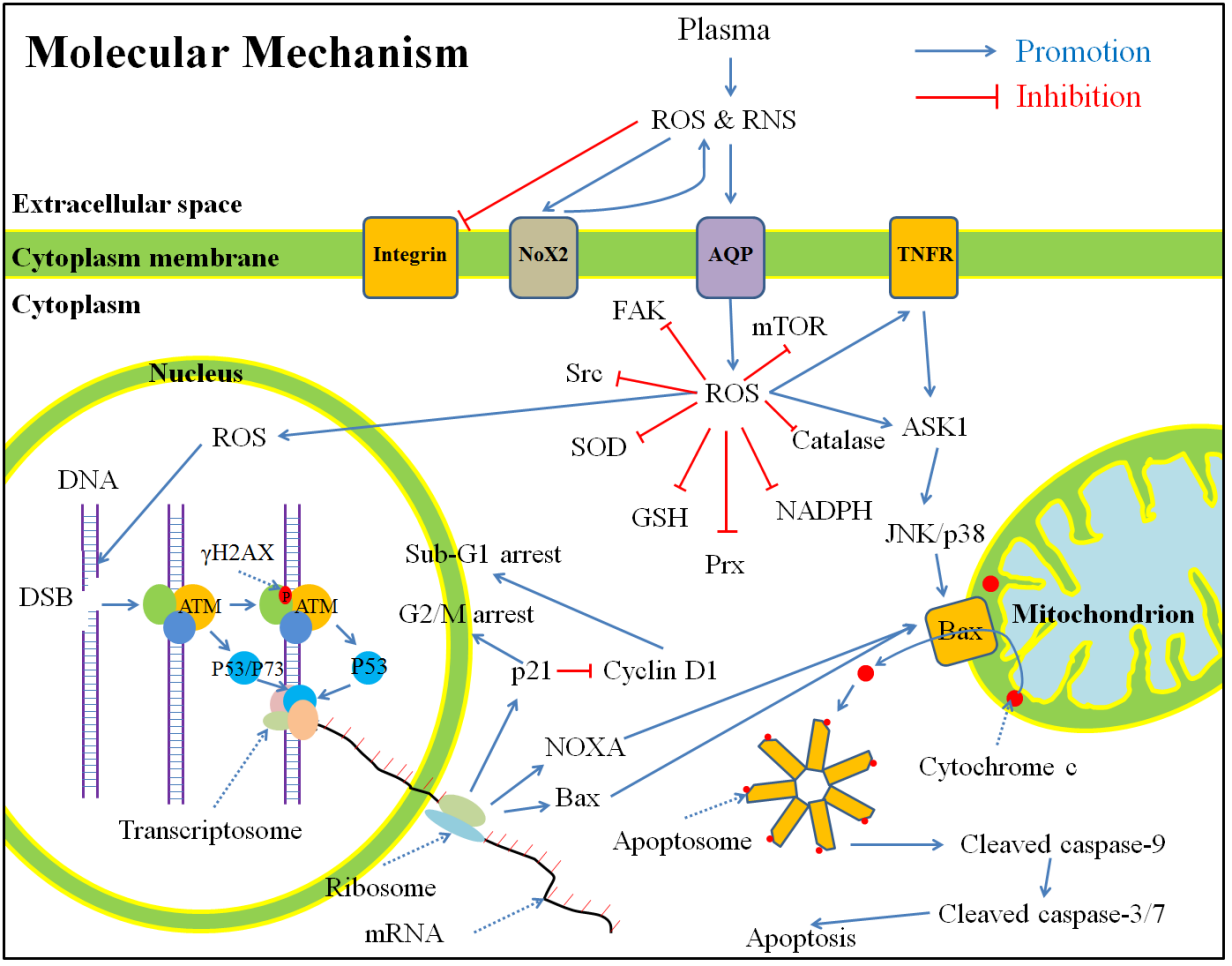
A general summary for the anti-cancer mechanism of CAP ***in vitro*** based on publications by the end of 2015. Shortly, the CAP-originated reactive species will cause a noticeable rise of intracellular ROS, which weakens the intracellular anti-oxidant system and further causes serious DNA double-strand break. As a result, cell cycle arrest and apoptosis based on mitochondrion-pathway or tumor necrosis factor receptor-pathway occur. Abbreviations: ROS: reactive oxygen species, RNS: reactive nitrogen species, Nox: NADPH oxidases, AQP: aquaporins, TNFR: tumor necrosis factor receptor, FAK, focal adhesion kinase, Src: Src kinase, SOD: superoxide dismutase, GSH: glutathione, Prx: peroxiredoxin, NADPH: reduced nicotinamide adenine dinucleotide phosphate, mTOR: mechanistic target of rapamycin, DNA: deoxyribonucleic acid, DSB: double-strand break, ATM: ataxia telangiectasia mutated, mRNA: messenger ribonucleic acid, ASK: apoptosis signal-regulating kinase, JNK: c-Jun N-terminal kinase.

To this end, primary understanding of the anti-cancer mechanism of CAP is obtained from *in vitro* studies. In this section, we present a summary based all references in this field by the end of 2015 (Figure 7). This summary does not tend to reflect the viewpoints of every researchers in this field, but to repesent the commonly acknowledged conclusions.

The CAP-originated ROS and RNS (directly from CAP or subsequently formed in medium) first touch the cytoplasmic membrane. Shortly after the CAP treatment with an adequately high dose, many cancer cells experience a morphological change from spread shapes to contractive shapes [2, 38, 49]. We observed this trend just 1 hour after the CAP treatment (unpublished results). In the contractive shape, the horizonal polarization and cytoplasmic protrusions are lost [104]. In addition, due to the appearance of tiny protuburances on the CAP-treated cancer cells [105], the cancer cells have a rougher surface than the untreated cancer cells [104]. The change of cell shape is accompanied with the architecture change of the cytoskeleton such as F-actin [2, 104, 106]. CAP treatment also decreases the expression of integrin [2, 29, 53], focal adhesion kinase (FAK) [2], Src kinase (Src) [104], and Rho family including Rho and Rac [104], which causes the commonly observed phenomenon that the CAP-treated cancer cells’ migration rate will be decreased and the cells’ detachment rate will be increased [29, 52].

### The transmembrane diffusion of reactive species

The significance of channels or transporters located in the cellular membrane has not yet been investigated in plasma medicine. However, these membrane proteins may be a key to understand the cellular response to the CAP treatment. Since most of plasma-originated reactive species are either charged or polar molecules, specific channels and transporters on the cytoplasmic membrane are necessary for the transmembrane diffusion of these reactive species (Table 1). So far, only NO has been widely regarded as a freely-diffusing molecule among other CAP-originated reactive species [17]. In fact, ONOO^-^ is also able to freely cross phospholipid membranes with a permeability coefficient 400 times greater than that of O_2_^-^ [107], though some unknown anion channels may also be involved in its transmembrane diffusion [108]. Other polar or charged reactive species, such as H_2_O_2_, NO_2_^-^ [109], and NO_3_^-^ [109, 110], must need channels or transporters to finish their transmembrane diffusion process. Aquaporins (AQPs), was first discovered as a specific water channel [111]. Recently, the key function of AQPs family in the transmembrane diffusion of several small molecules such as H_2_O_2_, CO_2_, NO, NH_3_, urea, and even glycerol has been confirmed [112]. Within the AQPs family, AQP 1, 3, 8, and 9 are the membrane channels facilitating the H_2_O_2_ transmembrane diffusion [113–116]. AQP 1 is even capable of facilitating the passive diffusion of NO across cell membranes [117]. Until recently, we first demonstrated the key function of AQP 8 in glioblastoma (U87MG) cells as the transmembrane diffusion channels of the cold plasma-originated H_2_O_2_. Silencing the expression of AQP 8 in U87MG cells significantly inhibits the anti-cancer capacity of the cold plasma-stimulated medium (unpublished results). Thus, AQPs may play be a key factor in the anti-cancer capacity of CAP particularly in terms of the transmembrane diffusion of the plasma-originated H_2_O_2_ [115]. Even while some authors proposed that the increased intracellular ROS was due to secondary products such as H_2_O_2_ from the plasma-activated NADPH oxidases (Nox) on the cytoplasmic membrane [53], the transmembrane diffusion of such secondary H_2_O_2_ is also rely on relied on AQPs [114, 118].

**Table 1:** The transmembrane diffusion patterns of some reactive species

Reactive species	Diffusion patterns	Channels/Transporters
NO	Passive diffusion/channels	AQP 1[117]
H_2_O_2_	Channels	AQP 1 [113], 3 [114], 8 [115], 9 [116]
NO_2_^-^	Transporters	NRT 2 [109]
NO_3_^-^	Channels/transporters	AQP 6 [110], NRT 1 [109], NRT 2 [109]
ONOO^-^	Channels	Unknown anion channels [108]

### The rise of intracellular reactive oxygen species (ROS) and redox balance

Significant rise of intracellular ROS is the most common response of the CAP-treated cells [119] including cancer cells [18, 120], normal untransformed mammalian cells [5, 18], and yeast cells [34]. Oxidant-sensitive fluorescent dye 2,7-dichlorodihydrofluorescein diacetate (H_2_DCFDA) is the most common intracellular ROS probes in this field [17, 22, 27, 56, 71, 120]. Because H_2_DCFDA is not a specific ROS probe, the chemical essence of increased intracellular ROS is still unknown. Nonetheless, many researchers proposed that H_2_O_2_ [56, 100, 120], OH. [51], and O_2_^-^ [81] were the main increased intracellular ROS in the CAP-treated cells. The significance of the increased intracellular ROS in the anti-cancer capacity of CAP has been widely verified through the observation that CAP cannot resist the growth of cancer cells if these cells have been pretreated with intracellular ROS scavengers such as N-Acetyl-Cysteine (NAC) [22, 27, 120], D-mannitol [120], rotenome [18], and apocynin [18]. For the normal untransformed mammalian cells, the pretreatment with NAC is also able to completely resist DNA double-strand breaks (DSB) due to CAP [5]. The origin of the increased intracellular ROS is still disputable. Most authors concur that it due to the diffusion of extracellular CAP-originated ROS across the cytoplasmic membrane [17, 18, 27]. This explanation is consistent with the observation that intracellular ROS rises just 5 min after the CAP treatment [17, 22]. Recently, Nox on the cytoplasmic membrane was found to cause the rise of intracellular ROS and the decreased viability of colorectal cancer cells after the CAP treatment [53]. The CAP-originated reactive species may just activate the expression of Nox2 in cancer cells but do not directly enter the cytoplasm [53]. Nox may generate secondary ROS such as superoxide, which further inhibits the cell growth [53]. Thus, the intracellular ROS may be partially due to the secondary ROS originated from oxidase on the cell cytoplasm membrane.

The disturbance on the intracellular redox balance is inhibited by diverse anti-oxidant systems, including small molecules such as glutathione (GSH), reduced nicotinamide adenine dinucleotide phosphate (NADPH) and ROS-scavenging enzymes such as superoxide dismutase (SOD), peroxiredoxin [121], catalase, thioredoxin reductase [122, 123], and glutathione reductase [124]. The rise of intracellular ROS will consume and weaken the inherent anti-oxidant system in cytoplasm. GSH, the most abundant small molecular thiol in mammalian cells [125], is an example. The reduced form of GSH is a tripeptide with a middle cysteine residue. Oxidation of the cysteine redisue forms a disulfide bond with a cysteine residue on another GSH. GSSG is used to denote the oxidized state of GSH [125]. The decreased GSH/GSSG ratio and NADPH/NADP^+^ ratio have been observed in the CAP-treated cancer cell lines [37, 96]. In addition, decreased expression of catalase [37], SOD [37], and peroxiredoxin (Prx) [126] has also been observed after the CAP treatment. Clearly, the weakened intracellular anti-oxidant system will facilitate the rise of intracellular ROS.

### DNA damage and apoptosis pathways

In the CAP-treated melanoma cells, the tumor necrosis factor receptor (TNFR)-based apoptosis pathway has been activated by the rise of intracellular ROS [127]. Similar apoptosis pathways have also been detected in the CAP-treated head and neck cancer cells [62]. However, most apoptosis pathways observed in the CAP-treated cancer cells is based on the mitochondrial pathway triggered by DNA damage and mitochondrial damage. DNA damage has been commonly observed as an early stage event upon the CAP treatment [44, 119]. DSB is the main damage style [27, 36, 64]. An important marker of DSB is a specific phosphorylation on serine 139 on H2AX histone (γ-H2AX), which has been commonly observed shortly after the CAP treatment [5, 27, 36, 38]. The serine 139 on H2AX is phosphorylated by ataxia telangiectasia mutated (ATM) recruited on the DSB site with other DNA damage response complexes [108, 128]. The enhanced expression of ATM has been observed in the CAP-treated oral cavity squamous cell carcinoma cells [129]. The activated monomeric ATM can phosphorylate p53 and other substrate in the DNA damage response complexes localized at DSB sites [129]. Phosphorylation of p53 (p-p53) has been observed in the CAP-treated oral cavity squamous cell carcinoma cells [129], and melanoma cells [130]. In addition, the increased expression of p-p53 in the CAP-treated mouse melanoma cells (B16F10) is just followed by the expression γ-H2AX [38], which provides clues to understand the chronological order of pathways. Moreover, ATM may also phosphorylate p73, which further activates the expression of pro-apoptotic factors [108]. The significant enhanced expression of p73 in the CAP-treated melanoma (Me1007) cells has been observed [131]. The knockout of p73 gene in Me1007 cells noticeably reduces the anti-cancer capacity of CAP [131].

The phosphorylation of p53 is a necessary step for a series of downstream cell cycle arrest pathways [108, 128]. The activation of p21 expression by p53 has been confirmed in diverse CAP-treated cancer cell lines [129, 130]. The activated p21 further triggers the different types of cell cycle arrests [132]. In the CAP-treated oral cavity squamous cell carcinoma cells, p21 inhibits the function of cyclin D1, causing noticeable sub-G1 arrest [129]. In the CAP-treated melanoma cells, p21 triggers G2/M arrest by an unknown pathway [130]. G2/M cell cycle arrest has also been observed in several other cold plasma-treated cancer cells [64, 133, 134], though the corresponding mechanism is unknown.

Moreover, phosphorylation of p53 is also necessary for triggering the mitochondrion-based apoptosis pathways [132]. p53 activates the expression of pro-apoptotic factors, such as Bax, PUMA, and NOXA [132]. These pro-apoptotic factors finally cause the release of cytochrome c and other intermembrane mitochondrial proteins into the cytosol [135, 136], where cytochrome c binds to apoptotic protease activating factor-1 (Apaf-1) and finally forms apoptosome [137]. The apoptosome further activates caspase-9 by cleavage [138, 139]. The activated caspase-9 further activates caspase-3/7 [138, 140] and finally starts a series of apoptotic events [138]. Among them, the cleavage of poly ADP-ribose polymerase (PARP) is an important early molecular marker of apoptosis [138]. Apoptosis is the main type of cancer cell death following the CAP treatment [43, 44, 119]. In the CAP-treated cancer cells, the release of cytochrome c into cytosol [38], the expression of NOXA [141], Bax [142], caspase-9 [143], caspase-3/7 [38, 47, 62, 130], the cleavage of PARP [31, 64], the loss of mitochondrial transmembrane potential [33, 38, 62, 96, 120], as well as DNA fragmentation [60] have been commonly observed. In short, the CAP-treated cancer cells not only follows the typical DNA damage pathways [108, 128], but also follows the well understood apoptosis pathways [144].

### Selective anti-cancer mechanism

In contrast to most anti-cancer methods such as chemotherapy and radiotherapy, the primary advantage of CAP is its selective anti-cancer capacity, which has been demonstrated over dozens of cancer cell lines [119]. Under the same experimental conditions, CAP tends to resist the growth of cancer cells rather than the growth of homologous normal cells [119] by triggering more apoptosis in cancer cells than normal cells [15, 16, 55]. Understanding such a selective anti-cancer mechanism is one of the key challenges in this field. Such a selective effect may be mainly due to the widely observed phenomenon that the noticeable rise of ROS selectively occurs in cancer cells rather than normal cells upon the same CAP treatment [18, 53, 96, 127, 145]. The measured ROS level in cancer cells is higher than that in normal cells after the CAP treatment [18, 53, 96, 127, 145]. Nonetheless, CAP kills more cancer cells than homologous normal cells in a few cases [133, 146].

To explain the observed trend, two models have been proposed. The first model proposes that such a different rise of ROS in the normal and cancerous tissue is due to different basal intracellular ROS levels between cancer cells and normal cells [9, 44]. Due to stronger metabolism in cancer cells, the basal ROS level in cancer cells is thought to be higher than that in normal cells [147–150]. When additional ROS stress such as the CAP-originated reactive species is exerted on cells, the whole intracellular ROS in cancer cells will pass a threshold more easily than that in normal cells [9, 44]. As a result, cancer cells experience more apoptosis than normal cells upon the CAP treatment [119]. However, this basal-ROS level model can just explain the appearance of higher ROS level in the CAP-treated cancer cells, but cannot explain the selective rise of ROS in cancer cells.

Recently, a novel model based on AQPs the only verified H_2_O_2_ channels on the cytoplasmic membrane has been proposed in a review [119]. Cancer biologists confirm that most cancer tissues tend to express more AQPs on their cytoplasmic membranes than homologous normal tissues [151]. After the CAP treatment, CAP-originated H_2_O_2_ diffuses into cancer cells significantly faster than homologous normal cells, causing a significantly higher rise of ROS in cancer cells than normal cells [119]. Despite the direct evidence for this model is still lacking, two indirect evidences support our proposition. First, the uptake of H_2_O_2_ in mammalian HEK 293 cells, human colon adenocarcinoma HT29 cells, as well as cervical cancer HeLa cells can be significantly increased by expression of AQP 3 on cellular membrane [114]. Second, the yeast cells expressing AQP 8 and AQP 1 show a much stronger resistance to H2O2 solution than the yeast cells do not express them [115]. Such different H_2_O_2_ consumption capacity between cancer cells and normal cells may be the foundation of the selective anti-cancer mechanism of CAP. The AQPs-based model not only explains the observed selective rise of ROS in cancer cells, but also explain our recent observation that glioblastoma cells tend to consume H2O2 in medium significantly faster than astrocytes [119]. It is necessary to emphasize that based on the AQPs-based model, the selective anti-cancer capacity of CAP treatment is not necessary to be exist. If cancer cells express less AQP 1, 3, 8, and 9 less than corresponding homologous normal cells, CAP may kill the cancer cells without selectivity or even kill more normal cells than cancer cells.

In addition to these models, an observation based on the correlation between the expression of p53 and the sensitivity of cancer cell lines to the CAP treatment has been reported [84]. It was found that the cells including cancer cells and normal cells expressing p53 gene were more resistant to the CAP treatment than cancer cells without p53 gene [84]. Before this finding, another trend that the cancer cells with a higher proliferation rate are more sensitive to the CAP treatment than the cancer cells with a lower proliferation rate has also been observed [106]. Loss of p53 is a key step during the tumorigenesis [75]. Many tumors in the high tumorigenic stage tend to lose p53 [75]. In addition, the cancer cells from a high tumorigenic stage tend to express more AQP than the cancer cells from a low tumorigenic stage. For example, the expression of AQP 8 in glioblastoma (grade IV astrocytoma) cells is higher than that in grade I, II, and III astrocytoma [152]. Though the direct correlation between the expression of p53 and AQPs has not been demonstrated in cancer biology, the AQPs-based model can theoretically explain the correlation between the expression level of p53 and the sensitivity of cancer cells to CAP. Simply, the cancer cells without p53 may tend to express more AQPs, making the cancer cells without p53 consume the CAP-originated reactive species such as H2O2 faster than the cancer cells with p53. This hypothesis might serve as a foundation for the p53-CAP sensitivity correlation.

**Figure 8:**
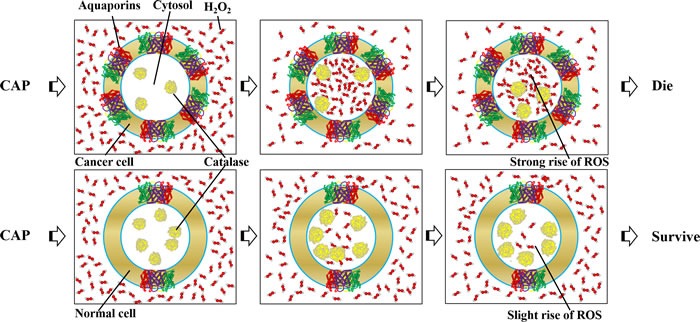
The modified selective model based on the distinct expression of AQPs and catalase in cancer cells and normal cells Cancer cells express more AQPs and less catalase than homologous normal cells in many cases.

The intracellular anti-oxidant system may also contribute to the selective anti-cancer mechanism, though the direct evidence is still lacking. The balance between the entrance of extracellularly originated ROS and the resistance from the intracellular anti-oxidant system determines the change of intracellular ROS [153, 154]. As mentioned above, the intracellular anti-oxidant systems include a series of enzymes such as catalase, superoxide dismutase, glutathione reductase, glutathione peroxidase as well as small molecules such as GSH and NADPH [155]. A very recent study demonstrated that the decreased expression and the enhanced expression of Cu, Zn-SOD or Mn-SOD enhanced and decreased the plasma-induced HeLa cell death, respectively [156]. Expression of exogenous catalase also blocked HeLa cell death [156]. Thus, a high expression of the intracellular anti-oxidant system may also weaken the anti-cancer capacity of CAP. Some specific anti-oxidant enzymes in cancer cell lines are indeed expressed less than corresponding normal cell lines [147]. Take the catalase as an example, its expression in cancer cells are less than the corresponding normal cells in many studies [147, 157–160]. However, a comprehensive review pointed out that the expression of catalase in other cancer cell line such as human H_2_O_2_-resistant HL-60 promyelocytic leukemia cell lines is higher than normal cell line [157]. The expression of catalase in gastric carcinoma cells is also higher than their non-cancerous counterparts [161]. Actually, even the conclusion that the cancer cells have weaker anti-oxidant systems than normal cells are still disputable in cancer biology [148]. For example, a strong Mn-SOD expression occurs in high-grade and advanced-stage bladder tumors [162]. In short, the selective anti-cancer capacity of CAP may be due to the combined effect of multiple cellular factors, such as the enhanced expression of AQPs as well as the decreased expression of specific anti-oxidant enzymes such as catalase in cancer cells (Figure 8).

## OUTLOOK

The application of CAP in the cancer treatment have been initiated just about 8 years ago. Thus, this is a very young field. So far, the research mainly focuses on the description of the anti-cancer effect of CAP *in vitro* and *in vivo*. The understanding of the anti-cancer mechanism is very limited. To become a mature anti-cancer treatment modality with a solid theoretical foundation, the basic mechanism needs to be addressed. There are many key questions have not been answered or even mentioned in this field. What is the essence of the selective anti-cancer capacity of CAP and what are the distinct cellular and molecular responses between cancer cells and normal cells to the CAP treatment? Similarly, which cancer subtypes are more or less sensitive to the CAP treatment? Can we find some general cellular and molecular features or markers to describe such killing selectivity? If we know the mechanism of such a distinction, can we enhance such a selectivity by controlling the chemical factors such as species generation as well as the physical factors such as the discharge in the CAP device? What is the contribution of physical factors such as the electromagnetic field to the anti-cancer capacity of CAP? In addition, for the clinical application, understanding the anti-cancer performance of CAP in animals is a key challenge. How to understand the inhibited growth of subcutaneous tumors just by a CAP treatment just above the skin? What is the potential toxicity of CAP? Can we eliminate the toxicity by optimizing CAP devices? What is the advantage of CAP compared with other existing treatment modalities? In our opinion, satisfactory answers to these questions will help CAP to become a practical anti-cancer tool.

## CONCLUDING REMARKS

In summary, CAP shows its promising potential to be a selective anti-cancer tool. Preliminary research over the past decade demonstrated that CAP could effectively inhibit the growth of dozens of cancer cell lines *in vitro* by mainly triggering apoptosis. CAP is also capable of effectively resisting the growth of subcutaneously implanted xenograft tumors in mice by unknown mechanism. The CAP-originated reactive species has been regarded as the primary factors resulting in cell death though physical factors of CAP may also has minor unknown functions. Despite the anti-cancer molecular mechanism is still far from clear, current studies reveal that the apoptosis of CAP-treated cancer cells *in vitro* is mainly due to the intense DNA double-strand break caused by a significant rise of intracellular ROS. The differential expression of the aquaporin channels and intracellular anti-oxidant enzymes such as catalases in cancer cells and normal cells may be a plausible mechanism to control the selective diffusion of reactive species across the cytoplasmic membrane of cancer cells and selective rise of intracellular ROS in cancer cells. Because corresponding homologous normal cells just experience a weak rise of ROS, CAP is able to selectively cause apoptosis in cancer cells *in vitro*.
